# Advances in monitoring mammalian circadian components and their rhythms using reporter systems†

**DOI:** 10.1039/d5cb00332f

**Published:** 2026-06-24

**Authors:** Bhavna Kalyanaraman, Emmanuel F. Rivera-Iglesias, Michelle E. Farkas

**Affiliations:** a Department of Chemistry, University of Massachusetts Amherst Amherst MA 01003 USA farkas@chem.umass.edu

## Abstract

Circadian rhythms are innate biological processes that cycle over periods of approximately 24 hours and are crucial in regulating many physiological and metabolic functions. At the molecular level in mammals, these cell-autonomous oscillations are controlled by a network of self-regulating feedback loops comprised of transcriptional and translational regulators. Reporter platforms for following promoter activity and/or proteins *via* detectable fluorescent and bioluminescent entities have facilitated the tracking of circadian elements in cells and organisms in real-time, which is especially important for studying such a dynamic biological system. As a result, reporters have been pivotal in uncovering fundamental aspects of the molecular core clock, changes in individual components in healthy *versus* various disease models, and screening for synthetic clock modulators. While the initially established tools of *in vivo* reporters (*i.e.*, Per2::Luc mice) continue to be widely utilized, newer ones have capabilities for evaluating other components and performing simultaneous assessments of multiple circadian components across different levels of expression. Concurrently, *in vitro* reporter models have been developed to track alterations in rhythms and protein presence in various cellular models, including those of disease. In this review, we discuss various reporter systems, including historical context, and their applications for monitoring rhythms at transcriptional and translational levels of expression, *in vitro* and *in*/*ex vivo*, with a focus on mammalian systems. Furthermore, we assess the advantages, disadvantages, and limitations of employing bioluminescent and fluorescent reporters in monitoring biological rhythms and components. Our goals are to provide an account of available reporter systems and pose considerations for their use and the development of technologies that can be used to expand our tracking and understanding of the molecular clock.

## Abstract 2

Circadian rhythms are innate biological processes, controlled by feedback loops; reporters have facilitated tracking of circadian elements in real-time. In this review, we discuss circadian reporter systems and their applications.

## Graphical abstract

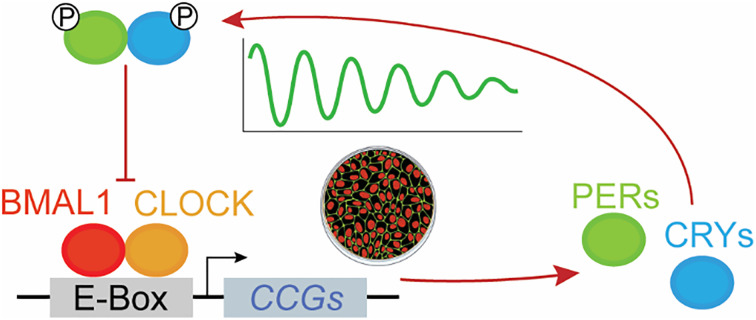

## Introduction

The circadian system in mammals is a highly complex network of cells and signaling molecules that form a hierarchy of central and peripheral oscillators. Together, these oscillators govern the circadian rhythms in organisms that are essential for their growth, development, and sustenance. At the heart of this elaborate system is the master clock, which lies in the suprachiasmatic nucleus (SCN) at the anterior part of the hypothalamus. The SCN is a tiny bilateral structure composed of approximately 20 000 neurons that receive direct photic input from the retina, generate self-sustained circadian oscillations, and entrain peripheral tissues.^1,2^ The widely accepted molecular clock model, described further below, provides a basic understanding of the interlocking feedback loops that govern daily circadian output in the SCN and peripheral tissues.

For decades, scientists have been investigating the molecular mechanisms of circadian rhythms. Mounting evidence indicates that the primary genes that form the basis of the circadian oscillator are conserved across species, from *Drosophila melanogaster* (Fruit fly) to *Mus musculus* (Mouse) to *Homo sapiens* (Humans).^3–5^ Extensive genetic, molecular, and biochemical approaches have provided insights toward understanding the circadian machinery in mammals.^6^ Circadian biology is by nature dynamic, and its regulation spans multiple levels of gene expression at different times of day. Experimental systems that permit the collection of data on the subtle changes in cyclic gene expression in a time-dependent manner are critical to studying circadian mechanisms.

The first report introducing circadian control of bioluminescence was in a marine dinoflagellate, *Gonyaulax*.^7^ This work described how the bioluminescent output could reflect the endogenous clock and helped shape the conceptual framework of circadian reporters. Since then, technological advances have enabled the selective monitoring of circadian gene expression using promoter and/or protein-driven expression of fluorescent and bioluminescent reporters. Some of the initial uses of luciferase reporters to track real-time oscillations in various organisms included cyanobacteria,^8^ fungi,^9^ plants,^10^ insects,^11^ rodents,^12,13^ and human cell lines.^14^ Of the earliest reports, a notable application of a bioluminescent reporter was in *D. melanogaster*, which pioneered monitoring of the temporally-regulated transcription of the clock gene *per*.^11^ Subsequently, this approach was employed to identify the presence of autonomous circadian oscillators throughout peripheral tissues that can be re-entrained.^15^ In addition to bioluminescent reporters, fluorescent reporters have also been used to track the expression of mammalian clock components. Some of the early work in this regard involved using green or yellow fluorescent proteins (GFP or YFP, respectively) to monitor the promoter activity of the murine *Per1* gene in tissue sections from transgenic mice^16^ and immortalized cell lines.^17^ In these promoter-reporters, the expression of the reporter gene is indirectly used to quantify and track the transcriptional activity of the circadian promoter over several days.

Reporter systems provide flexibility to use different tracking entities to monitor the dynamic circadian system over multiple cycles, with frequent sampling permitted in many instances. Based on the large amounts of data they provide, reporters enable detailed assessments of various circadian parameters, including amplitude, period, and phase, and in some cases can yield information on the spatiotemporal dynamics of individual components. In contrast, mRNA and protein expression levels are often directly tracked using reverse transcriptase PCR (RT-PCR) and western blotting, respectively. More recently, genome-wide analyses using next-generation sequencing (NGS) techniques like chromatin immunoprecipitation (ChIP-seq) and RNA sequencing (RNA-seq) have widened the ability to monitor the expression, regulation, and stability of circadian mRNA.^18–20^ These approaches have provided many important insights into the transcriptional regulation of core clock genes (described in the following section), including *Bmal1*.^21,22^ While these types of assessments can provide data across numerous genes and transcripts simultaneously, they are expensive. Limitations of these non-reporter assays also include that they are not amenable to frequent sampling (and, hence, cannot reliably be used to extract detailed information on circadian oscillations or detect subtle changes therein), and in most cases, invasive procedures must be used, or euthanasia performed for *in vivo* experiments, and cells lysed for *in vitro* studies, meaning that sampling occurs independently and not from the same biological specimen. Animal models and cell lines that express the reporter of choice fused to or driven by a circadian promoter/protein circumvent these issues, as tracking can occur frequently and from the same entity. Hence, RT-PCR, western blotting, and NGS can be used together with reporter-tracking studies to provide a more complete understanding of cyclical changes in clock genes.

The development of reporter systems has been pivotal in tracking circadian components and understanding their roles *via* perturbation studies (*e.g.*, genetic knockdown, overexpression, and treatment with chemical entities).^21,23–26^ Numerous reporter systems have been developed to track circadian components at the promoter/transcriptional and protein levels, including specific paralogs or isoforms *in vivo*^27–29^ and *in vitro*,^30,31^ to better understand their roles in the circadian machinery and timing. These tools have been important in gaining new insights about core clock entities, changes that occur with disease or particular stimuli,^32,33^ and the identification and characterization of circadian clock effectors (*e.g.*, small molecules or other agents).^34,35^ More recently developed reporter platforms, including those that enable the concurrent tracking of multiple components,^28,36^ continue to be developed and enhance our knowledge of the circadian system. The advantages of reporter-based tracking include the ability to monitor transcriptional and translational expression in a high-throughput manner, with frequent sampling, lack of perturbation to the biological system during tracking, ease of reproducing experiments, and minimization of human error (*e.g.*, pipetting).

Here, we provide information on breakthroughs in and reporters available for tracking mammalian rhythms at the transcriptional and translational levels *in*/*ex vivo* and *in vitro*, shortfalls of currently available reporter-tracking systems, and possible avenues for future research in this area. We also provide primers on the circadian clock (including molecular mechanisms) and reporter systems for reference.

## Circadian mechanism in mammals

In mammals, the circadian machinery can be classified into three tiers—the input pathway, the central oscillator (or SCN), and the output pathway.^37^ Various input cues, including light as the dominant signal, entrain the SCN *via* circadian photoreceptors. Individual SCN neurons are stable, self-sustained circadian oscillators that can generate and restore behavioral rhythms.^38^ While SCN neurons do not rely on intercellular coupling to generate circadian rhythms, the network interactions restrict the range of expressed periods and phases, thereby resulting in robust, synchronized oscillations.^39–43^ The tightly coupled SCN oscillators subsequently entrain peripheral oscillators that include clock-controlled genes (CCGs) in various tissues, including the heart, lung, kidneys, spleen, pancreas, and other peripheral organs.^27,44,45^ The SCN entrains the peripheral clock to generate daily circadian outputs with the help of direct cues like humoral or sympathetic nerve signaling and indirect cues, including rest-activity cycles, body temperature, or feeding.^46^ However, owing to differences in the rigidity and intercellular coupling of the peripheral cells to the SCN, changes in circadian parameters are far more varied in peripheral cells.^39^

**Fig. 1:**
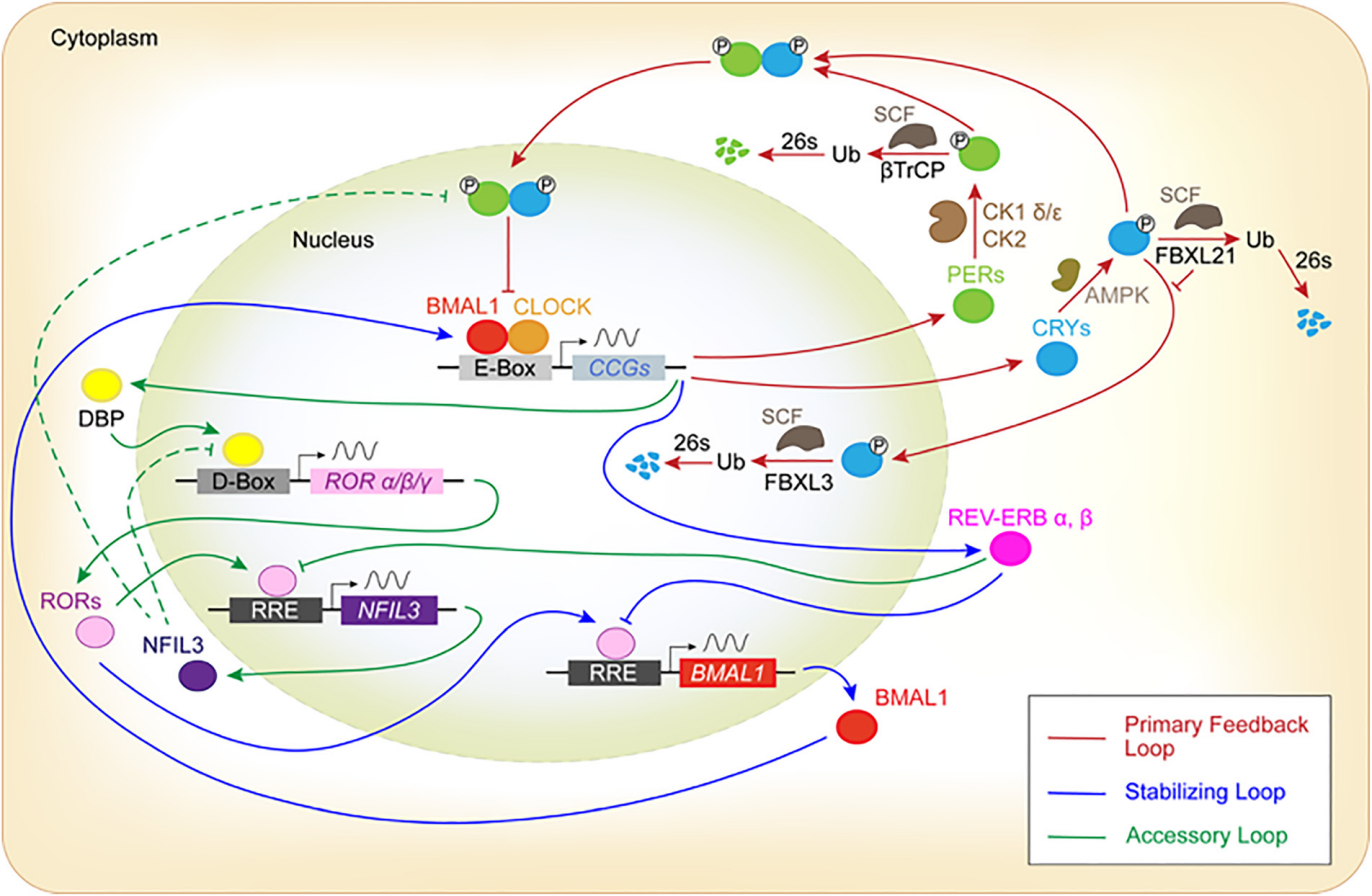
Molecular mechanism of the mammalian core circadian clock. The primary TTFL (red) includes the BMAL1:CLOCK heterodimer, which binds to E-boxes and initiates the transcription of CCGs including PER and CRY. PER and CRY form a heterodimer and bind to BMAL1:CLOCK to regulate their own expression. Concurrently, PER and CRY levels are regulated by kinases CK1(δ,ε)/2 and AMPK respectively, leading to ubiquitination (Ub) by β-TrCP 1/2 and FBXL 3/21, and subsequent degradation by the 26S proteasome. The stabilizing loop (blue) is formed by the REV-ERBs and RORs that bind to the REV-ERB-ROR response element (RRE), and regulate the expression of BMAL1, thereby regulating the primary loop. The accessory loop (green) includes DBP protein, which binds to the D-Box and initiates the expression of RORs and subsequently NFIL3. NFIL3 further regulates the accessory loop by repressing DBP and the primary loop by repressing the activity of PER2 proteins.

At the molecular level, SCN neurons, as well as peripheral cells, have a cell-autonomous circadian clock governed by an autoregulatory transcription-translational feedback loop (TTFL) (Fig. 1). The primary loop of the TTFL is comprised of four core clock genes that form an interlocking network: brain and muscle arnt-like 1 (BMAL1), circadian locomotor output cycles kaput (CLOCK), period (PER), and cryptochrome (CRY).^47^ BMAL1 and CLOCK are transcriptional activators that heterodimerize, bind to promoter E-box enhancer regions (5′-CANNTG-3′), and activate the transcription of *PER* (1/2/3), *CRY* (1/2), and CCGs in the positive arm of the feedback loop.^48^ On the other hand, PER and CRY proteins are transcriptional repressors that are translated and accumulate in the cytoplasm. Upon reaching threshold concentrations, PER and CRY heterodimerize, translocate into the nucleus, and bind to the BMAL1-CLOCK heterodimer, thereby blocking its enhancer activity as the negative feedback loop.^49,50^ The cycle is re-established once the levels of PER and CRY have dropped below their threshold concentrations. The entire feedback cycle takes approximately 24 hours. In addition to the primary feedback loop, the BMAL1-CLOCK heterodimer activates the transcription of nuclear receptors REV-ERB α and β (REV-ERBα/β). The REV-ERBs compete with the retinoid orphan receptors α, β, and γ (RORα/β/γ) to bind to the REV-ERB-ROR response elements (RREs) present in the enhancer regions of the *BMAL1* promoter, thereby promoting *BMAL1* expression.^51,52^ A third accessory loop also exists, which involves BMAL1-CLOCK-mediated regulation of RORs *via* D-site binding protein (DBP).^53,54^

In summary, the molecular clock is regulated by DNA *cis*-regulatory elements, including E-boxes, D-boxes, and RREs, which exhibit bidirectional control over the interlocking feedback loops, mediated by many activators and repressors.^48,50,55–59^ As repressors play a crucial role in determining circadian parameters, studies have focused on developing tools to closely monitor their expression patterns in health and disease while also tracking the downstream effects of modulating their expression. Also, the presence of several self-regulating clock components and secondary regulatory networks makes it pertinent to appreciate the complex hierarchical nature of the mammalian circadian machinery, with the need to reinterpret the established model using analyses at multiple levels of gene expression.^60^ In addition to utilizing standard assays to quantify circadian mRNA and proteins, reporters as chemical biology tools have been developed and used to track clock components and/or their oscillations at the promoter and protein levels. Using this technology, scientists have been able to study the role(s) of individual circadian paralog/isoforms and their interactions with each other; they have also been used to track their spatial localization over the period of a circadian cycle.

## Technological advances in long-term monitoring of circadian components

The transcriptional, translational, and regulatory roles of clock components follow a complex time- and inter-dependent pattern that requires multi-faceted and dynamic chemical biology tools to be able to monitor them. The development of transcriptional and translational reporters has helped to elucidate many connections between the expression of circadian components and changes in their rhythmic patterns due to downstream regulatory processes at the cellular level. This information is critical to understanding the roles and effects of circadian genes and proteins in health and disease. A key advantage of using circadian reporters is that the signal output can be directly used to probe for cell autonomous, self-sustained oscillations within an *ex vivo* tissue explant or in *in vitro* cell models. In contrast, conventional population sampling methods (*i.e.*, periodic tissue sampling and mRNA/protein quantification) cannot distinguish whether the observed rhythmicity is a result of systemic cues (*e.g.*, SCN entrainment cues, feeding cycles, *etc.*) or due to an inherent clock.

**Fig. 2:**
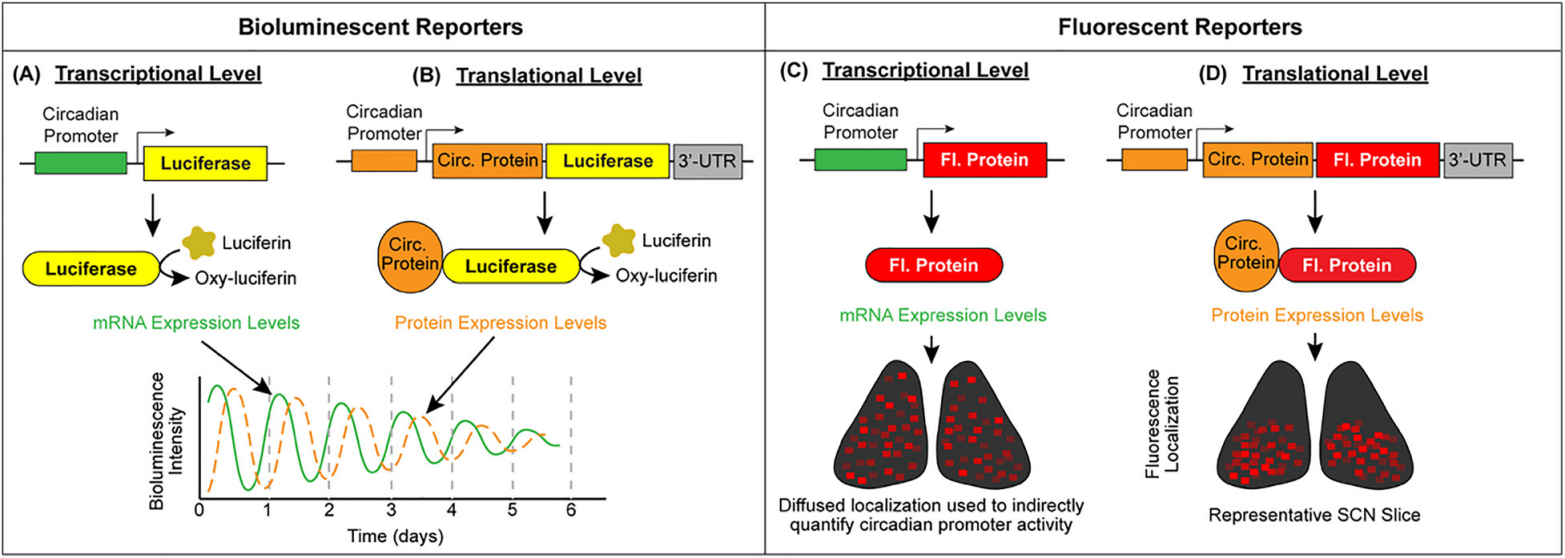
Design strategy of bioluminescent and fluorescent reporters. (A) Transcriptional-level bioluminescent reporters are used to monitor mRNA expression levels indirectly by tracking luminescence from the luciferase reporter under the control of the circadian promoter. The oscillations can be monitored in real-time over several days to study circadian parameters, including amplitude, period, and phase. (B) Translational bioluminescent reporters track protein expression by generating a fusion circadian protein. Due to the delay caused by the translation of the circadian mRNA, protein level oscillations show a significant phase delay when compared with transcriptional level oscillations. (C) The design of transcriptional fluorescent reporters is similar to their bioluminescent counterparts, wherein the fluorescent protein is expressed under the activity of a circadian promoter; this reporter cannot be used for spatiotemporal imaging. (D) Translational fluorescent reporters involve a fluorescent fusion circadian protein that can be used to visualize the spatiotemporal localization of the circadian protein in tissue explants (shown) and/or individual cells.

Circadian reporter systems should possess certain features to accurately display rhythmic dynamics with sufficient temporal resolution. One of the main considerations in reporter design is turnover rate. Optimal circadian reporters exhibit short half-lives, intrinsically or engineered through destabilization strategies (*e.g.*, PEST-tagged luciferases).^61,62^ In addition to these features, reporters should also exhibit a high signal-to-noise ratio, low background, and high sensitivity.^63^ Reporters in circadian biology can be classified based on the nature of the circadian component being tracked, and are typically transcriptional (promoter-based) or translational (protein-level) reporters; other types are briefly described at the end of this section. In a transcriptional reporter construct, the circadian promoter drives the expression of a bioluminescent or fluorescent entity, which can then be imaged by treating with d-luciferin (or other) substrate to produce bioluminescence or excited to generate fluorescence, respectively (Fig. 2a and c). Transcriptional reporters can be generated by integrating the reporter into the endogenous circadian promoter region^27,64^ or by using full-length or truncated portions of the promoter to generate a promoter-reporter plasmid construct.^30,65,66^ Endogenous incorporation of the reporter can be used to understand the fundamental transcriptional nature of the circadian promoter, including the role of the enhancers and repressors in the promoter sequence. Conversely, truncated promoter-reporters are suitable for broadly monitoring rhythmic activity and high-throughput screening studies, as they retain the minimal promoter region that can generate robust, reproducible oscillations and can be employed in a variety of models. A translational reporter is generated by inserting the luciferase or fluorescent protein sequence at the C- or N-terminus of the circadian protein using flexible linkers like glycine–serine linkers (GlyGlySer)_*n*_, where *n* is the number of repeats (Fig. 2b and d).^67^ The C-terminus is typically used for reporter fusion to preserve protein function and dynamics.^68,69^ This results in the expression of a fusion protein; specifically, the traceable entity is only produced with expression of the protein of interest. While a reporter-fused protein can facilitate monitoring of the dynamics of the circadian component, a potential limitation is that the fused reporter entity could affect the protein's normal functioning.^70^ Use of T2A linker sequences addresses this limitation by facilitating the translation of the native circadian protein and the reporter entity independently of one another.^62^

Animals and cells expressing the recombinant promoter or protein reporters can be produced in different ways, depending on the nature of the gene fragment. Transgenic manipulation has been at the forefront of generating reporters for understanding the functioning of the mammalian circadian clock *in*/*ex vivo* and *in vitro*. Plasmid constructs containing transgenes of specific circadian components and the selected reporter can be incorporated into animal models, primary cells from tissue explants (*ex vivo*) or immortalized cell lines (*in vitro*).^71–73^ The exogenous gene expression cassettes can be incorporated into cells by transient transfection, stable transfection, or CRISPR (clustered regularly interspaced short palindromic repeats). Transient transfection mediated by transfection agents (lipofectamine), cationic polymers (*e.g.*, polyethylimine), or non-chemical methods (electroporation) promotes short-term expression of the “clock-controlled” reporter and is often used in *in vitro* cell culture models.^13,59,74^ Stable transfection using viral vectors is more efficient and allows for integration of the exogenous cassette into the genomes of both dividing and non-dividing cells, facilitating long term expression over multiple passages. Bacterial artificial chromosome (BAC) libraries and transduction with adenoviruses (or adeno-associated viruses, AAVs) are commonly used to express circadian reporter systems *in vivo*.^27,75^ Organ and tissue explants from animal reporter models can then be cultured for further studies. Viral (AAV, adeno/lentiviral) transfections are standard means to transduce *ex vivo* organotypic tissue slices, explants, or organoids and *in vitro* models. However, the insertion location is largely random.^76^ Using CRISPR-based gene editing, the reporter sequence is inserted at the genomic loci of the target circadian gene, allowing for precise gene editing at the targeted site with reduced impact on genomic stability and homeostasis.

Reporter systems also can be broadly classified into bioluminescent and fluorescent types, depending on the detectable entity used. Reporters have been widely used for long-term monitoring of circadian oscillations in live animals, explants, and cells (*in*/*ex vivo* and *in vitro*, respectively) by tracking signals associated with elements of the core clock.^13,62,77^ Luciferase/bioluminescent reporters work *via* expression of a functional luciferase enzyme under the control of the promoter or protein of the gene of interest. A d-luciferin/coelenterazine (or synthetic derivative) substrate is added to the sample, and undergoes oxidation in the presence of the luciferase enzyme, resulting in bioluminescence.^63^ The type of substrate required is determined by the nature/source of the luciferase gene/enzyme used. Substrates’ chemical and physical properties vary, with differing stabilities, solubilities, and kinetics, thereby affecting the reporter's signal and reproducibility.^78,79^ For example, coelenterazine can be coupled with *Renilla*, *Gaussia*, and related luciferases; however, due to its hydrophobicity and tendency to auto-oxidize, it has limited scope compared to synthetic analogues or derivatives.^80,81^ Another aspect to consider is substrate availability, as its reaction is the rate-limiting step in generating signal.^82^ This is particularly challenging in long-term *in vivo* circadian experiments. There are multiple approaches to substrate administration *in vivo*; the most commonly used are injections,^80^ drinking water supplementation,^73^ and continuous administration *via* osmotic pumps.^83^ Fluorescent proteins like GFP are expressed under the control of a circadian promoter or fused to the circadian protein by molecular cloning or CRISPR. The tagged fluorescent proteins can be excited at specific excitation wavelengths corresponding to the fluorescent protein to obtain fluorescence output.^63^

Fluorescent reporters are often used to track the spatiotemporal dynamics of circadian proteins. These systems have been reported to have brighter outputs with better resolution than luminescent reporters. They can facilitate an array of chemical biology studies, including visualizing and quantifying spatial dynamics, (*e.g.*, tracking nuclear *versus* cytosolic localization of circadian clock proteins within single cells), protein-protein interactions, assembly formation, abundance, and stoichiometry;^28,68,84,85^ for further information, we refer the reader to a recent review.^86^ However, fluorescent reporters have significant limitations in long-term, frequent monitoring due to photobleaching and lower sensitivity due to autofluorescence. While shorter durations (60–72 hours) are sufficient for visualizing oscillations,^87,88^ they do not provide enough data points to accurately determine circadian parameters. This can be addressed by using brighter fluorescent probes and tuning exposure times.^75,77^ In addition, the maturation times and half-lives of fluorescent proteins vary depending on the nature of the fluorescent protein used, which needs to be scaled appropriately to obtain accurate measurements.

In contrast, bioluminescent reporters allow long-term imaging with frequent sampling in the presence of sufficient substrate concentration. They allow tracking of subtle changes in circadian oscillations due to the shorter half-lives of luciferase enzymes typically used, and the reporters are highly sensitive and generate luminescence signals at extremely low expression levels. Luciferase-based reporters are often used in phenotypic and endpoint circadian-based studies to discover and test modulators of clock function. These assays are generally cell-based and have been coupled with high-throughput screening approaches, enabling the interrogation of large chemical and molecular libraries. Using such approaches, RNAi-based and small-molecule screens have identified modulators that alter circadian parameters, including period, phase, and amplitude, as well as impact circadian regulatory pathways.^89–94^ However, with some exceptions,^95,96^ bioluminescent reporters are limited to assessment of population-level dynamics. While bioluminescence imaging (BLI) can be used to monitor the spatial localization of these signals, their lower intensity and higher attenuation result in poor resolution. Another consideration in implementing luciferase-based reporters to study circadian oscillations is that their activity depends on intracellular ATP, oxygen, pH, and the cellular metabolic state.^97^ Hence, outputs could be affected by these parameters. For example, it has been reported that in confluent, non-dividing cell cultures, luciferase reporters can reflect metabolic dynamics, such as ultradian oscillations, which are usually lower in amplitude than canonical circadian rhythms.^98^

Table 1 lists common bioluminescent and fluorescent reporter probes that have been used for monitoring core clock components, their approximate sizes, relative brightnesses, and sensitivities. A previous review sheds further light on substrate requirements, half-lives, and spectral overlap in reporter design.^63^ Taking the advantages and disadvantages of these reporters into consideration, scientists have developed various bioluminescent and fluorescent reporter systems to track several core circadian components at different levels of expression *in*/*ex vivo* and *in vitro.*

**Table 1:** Common reporters, their characteristics, and uses in circadian tracking

Reporter class (References)	Approx size (kDa)	Examples	Brightness	Approx. half-life (h)	Advantages/applications	Limitations/caveats	Feasible models for use
Beetle luciferases^27,99–101^	61–63	Firefly luciferase (Fluc), Click beetle luciferases (CBR/CBG)	High	3–4	High SNR	Limited protein spatial information compared to fluorescent reporters	*In*/*ex vivo*, *in vitro*
					Excellent for long-term recording		
Renilla luciferase^a^^102^	36	Renilla luciferase (Rluc)	Medium	3–5	Medium SNR	Lower brightness compared to beetle luciferases	*Ex vivo*, *in vitro*
					Widely used in dual reporter assays		
Nano-luciferase^70^	19	NanoLuc	Very high	>15	High SNR	Less validated in studying molecular circadian systems relative to firefly luciferase systems	*Ex vivo*, *in vitro*
					Excellent for detecting low expression genes		
Secreted luciferases^103^	19–62	*Gaussia* luciferase (Gluc), *Cypridina* luciferase (Cluc)	High	>24	Medium SNR	Secreted signals tend to accumulate and do not provide high-fidelity real-time circadian dynamics	*In vitro*
					Allows media sampling media without disturbing culture		
Fluorescent proteins (FPs)^75,77,84,104^	27–29	EGFP, YFP, mCherry, mClover, mScarlet	Medium	>24	Low SNR	Potential photobleaching and phototoxicity in longer duration experiments	*In*/*ex vivo*, *in vitro*
					Best for imaging spatio-temporal dynamics		
BRET systems^a^^102^	50–60	NanoLuc-FP systems	High	Variable^b^	High SNR	Signal depends on many factors: donor/acceptor stoichiometry distance, and geometry	*Ex vivo*, *in vitro*
					Complements luciferase reporters to gain mechanistic insights		

a This system utilized cyanobacteria, no current mammalian examples exist.

b The overall stability of BRET signals depends on the intrinsic half-lives of the donor and acceptor.

We note here that while most reporters follow transcriptional or translational outputs, the circadian clock also extends to processes including intracellular signalling pathways, membrane excitability, and intercellular communications, which require other systems for tracking.^105,106^ The implementation of luciferase-based biosensors, Okiluc-aCT and Okiluc-CaM, enabled tracking of intracellular messenger cyclic AMP (cAMP) and Ca^2+^, respectively, in cells, providing evidence that its rhythms are independent yet phase-coupled to core clock gene expression.^107^ Genetically encoded neurotransmitter sensors for extracellular glutamate and GABA have been used in circadian research, including iGluSnFR and iGABASnFR, respectively.^108,109^ For example, the iGluSnFR system was used to reveal that astrocytes actively set and maintain circadian tempo in the SCN,^110^ and iGABASnFR helped determine that this occurs *via* astrocytes’ control of rhythmic GABA levels.^111^ Ionic signaling also represents a vital layer of circadian regulation. Use of a genetically encoded calcium indicator variant of GCaMP3 uncovered oscillations in intracellular Ca^2+^ and was used to demonstrate that Gq-mediated Ca^2+^ signaling and VIP activation are required for clock synchronization in SCN neurons.^112^ Beyond GCaMP3, there is an entire ecosystem of genetically encoded sensors that can detect many different ions, including Cl^−^, K^+^, Na^+^, H^+^, Zn^2+^, and Mg^2+^; we refer readers to other resources for additional information.^113^ In addition to intracellular and ionic signaling, encoded sensors can measure differences in membrane voltage potential (*i.e.*, ArcLightD). This system was implemented in SCN neurons and revealed that electrical activity exhibits robust, network-synchronous oscillations that differ and are partially coupled to the intracellular Ca^2+^ circadian rhythm.^114,115^

### Reporters to assess circadian rhythms *in* and *ex vivo*

The interest in understanding mammalian rhythms, especially in rodents, has taken precedence due to their importance as models for human physiology.^116,117^ Over the past several years, multiple mouse models have been generated to understand the complex hierarchical oscillators at many levels.^118,119^ Transgenic mice carrying luciferase genes driven by circadian promoters designed to examine circadian oscillations at the transcriptional level were among the first *in vivo* reporters used to study circadian oscillations. Using these models, it was established that the luminescence from the firefly luciferase gene reflects the mRNA rhythms of the circadian gene using real-time optical imaging.^71^ Other studies have employed clock-driven reporters to study the disruption of clock components in *in vivo* disease models for diabetes,^120^ neurological disorders,^121^ and metabolic disorders.^122,123^

In the early 2000s, researchers focused on generating firefly luciferase reporters using truncated murine *Per1* promoters. These studies demonstrated that variations in promoter length and region directly influenced promoter activity, thereby highlighting the presence of multiple regulatory regions in the promoter sequence.^48,124,125^

*Per1* promoter reporters also showed dampening of *Per1* promoter activity in the SCN and peripheral tissues.^66,71,73,126,127^ This finding was challenged following the generation of PER2:Luciferase (Luc) mice by knocking firefly luciferase (FLuc) into the exogenous C-terminus of the *Per2* gene. PER2:Luc mice made it possible to track mPER2 protein oscillations *in vivo*, while also indicating that paralog-dependent variations and post-transcriptional regulations were involved.^27^ Despite the subsequent dampening of PER2:Luc protein reporter amplitudes, self-sustained oscillations (>20 cycles) were reported in peripheral tissue explants from the transgenic mice. To date, this reporter is the most frequently used for *in* and *ex vivo* tracking experiments. Tissue explants and cells isolated from PER2:Luc mice have been used to investigate the interdependence of core circadian components in the SCN^87,128^ and to analyze circadian rhythmicity in peripheral tissues.^99,129^ As extensions of the sustained rhythms finding, studies showed that dampened PER2:Luc protein rhythms were present in peripheral tissues for almost a month in SCN-lesioned (SCNx) mice kept in constant darkness.^99^ These findings highlighted the crucial role of reporters in determining the self-sustained oscillations of the peripheral clocks in the absence of a master synchronizer. Other studies using explants from PER2:Luc mice validated the presence of small independent rhythms in peripheral cells controlled by the SCN.^96,99,128,129^ While *ex vivo* models are used complementarily to *in vivo* models to monitor circadian properties, explantation procedures and culture conditions can reset tissue clocks.^130,131^ This serves as a potential pitfall since the phase of the *ex vivo* explants will no longer reflect the *in vivo* phase. This limitation can be addressed by collecting explants at multiple time points to assess the effect of explantation timing on circadian phase measurements.^131,132^

Different iterations of *in vivo* Bmal1 promoter-reporters have been generated with varying promoter lengths and different luciferase gene fragments.^133,134^ Work involving these reporters was among the first that introduced the possibility of simultaneous tracking of multiple core clock components, such as tracking *Bmal1*-Eluc and *Per2*-FLuc.^100^ The systems used enhanced beetle luciferase (Eluc) and firefly luciferase (Fluc), which required d-luciferin substrate and generated luminescence outputs at different wavelengths. While Per1/2 and Bmal1 reporters have been widely used at both promoter and protein levels, in the past decade, there has been a steady increase in the generation of Cry reporters and reporters to track non-core clock components involved in circadian timekeeping.^28,62,135^Table 2 summarizes some significant transcriptional and translational core clock reporters used for circadian studies both *in vivo* and *ex vivo*.

**Table 2:** Reporters used to track core clock components *in vivo* and *ex vivo*

Reporter (Original Reference)	Type	Detectable entity	Uses/adaptations (Citations)
Per1^125^	Promoter	Firefly Luc	*In vivo* ^71,72,136^
Per1^a^^124^	Promoter	Firefly Luc	*In vivo* ^73,129,137^
Per1^66^	Promoter	Firefly Luc	—
Per1^127^	Promoter	Firefly Luc	*In vivo* ^138,139^
Per1^140^	Promoter	*Vargula hilgendorfii* Luc	—

Per2^27^	Protein	Firefly Luc	*In vivo* ^84,87,96,99,127,128,139,141–143^
Per2^77^	Protein	Venus	*Ex vivo* ^142^
Per2^144^	Protein	mVenus/mRuby3	—
Per2^74^	Promoter	Firefly Luc	*Ex vivo* ^141^
Per2^143^	Promoter	Firefly Luc	—
Per2^75^	Promoter	Venus	—
Per2^36^	Protein	Red and Green Beetle Luc	—

Bmal1^140^	Promoter	Firefly Luc	—
Bmal1^b^^133^	Promoter	Red Beetle Luc	*In vivo* ^145^
Bmal1^134^	Promoter	Enhanced Luc	*In vivo* ^100,138,139^
Bmal1^143^	Promoter	Firefly Luc	*Ex vivo* ^141^
Bmal1^146^	Promoter	Firefly Luc	*Ex vivo* ^147^
Bmal1^142^	Protein	Venus	*Ex vivo* ^28^

Cry1^87^	Promoter	Firefly Luc	—
Cry1^75^	Promoter	Venus^c^	*In*/*ex vivo*^135^
Cry1^28^	Protein	mRuby	—

a This article introduced the mPer1 promoter-reporter for *in vitro* studies (NIH-3T3 cells); it was eventually also used for *in vivo* studies.

b This reporter uses the rat Bmal1 promoter sequence; all others use murine.

c The reporter's signal was detected using an optical fiber, surgically inserted into a mouse brain SCN.

In addition to using reporters to monitor the expression of individual components, some studies have used a combination of different reporter systems to facilitate dual-component tracking. The first account of dual-tracking allowed the monitoring of two murine clock genes, *Per1* and *Bmal1*, from a single peripheral tissue.^140^ The murine *Bmal1* promoter was fused with FLuc, while the *Per1* promoter was fused with *Vargula hilgendorfii* luciferase (VLuc), a secreted protein. *Bmal1*:FLuc was detected in real-time, and the expression of *Per1* was sequentially monitored by quantifying the VLuc secreted into the media. While this dual reporter system successfully demonstrated self-sustained oscillations in peripheral tissues, there is a real limitation in the reliability and consistency of Vluc quantifications. In a different study, enhanced beetle luciferase (ELuc) was introduced to monitor *Bmal1* promoter expression *in vivo*,^134^ and has been used in tandem with the *Per1* promoter^139^ and mPER2 protein luciferase reporters.^99,139^

*In vivo* fluorescent protein reporters have also been developed and utilized, including in the successful tracking of mPER2:Venus spatiotemporal dynamics, and the interactions of mPER2:Venus with mCRY1:mRuby3 and mCRY2:EGFP in *ex vivo* tissue models.^112^ Using fluorescence imaging, it was revealed that CRY proteins dose-dependently control the nuclear localization of PER2 by using their C-terminal tail, thereby regulating the circadian period in *ex vivo* SCN slices.^142^ Recently, Smyllie *et al.* reported a quantitative analysis of the relative abundance, stability, and spatiotemporal dynamics of PER2, CRY1, and BMAL1 in mouse SCN slices by using a PER2 color-switch platform to compare protein levels across two different fluorescent protein reporters.^144^ This system facilitated accurate stoichiometry assessments between core circadian proteins and comparison with previous studies using knock-in fluorescent reporter mice. Their findings demonstrated that PER2 and CRY1 do not temporally coincide to form the transcriptional repression complex, as previously assumed. Further, the relative abundances and stabilities of PER2, CRY1, and BMAL1 differ, with PER2 being the limiting factor relative to CRY1 and BMAL1 baseline levels.

### *In vitro* reporters and experiments to track mammalian circadian rhythms and components

*In vitro* reporter models have been very useful in providing insights into variations in circadian expression patterns across different cellular types and disease models. While *ex vivo* reporters also permit the tracking of circadian behavior in peripheral tissues, they require the isolation of primary cells from animals, which requires use and maintenance of the animals themselves; these cells are also typically non-proliferative and techniques to use them can be complex and labor-intensive. *In vitro* reporter models, on the other hand, use immortalized cell lines that are easier to handle and can be used to study a wider range of cell types with less infrastructure needed. The ease of handling immortalized cell lines and manipulating them to express circadian reporters has resulted in using *in vitro* reporters as screening tools to probe factors and entities that may regulate and/or affect circadian oscillations.^89,148,149^ Reporters serve as the most reliable tool to facilitate high-throughput screening (HTS) for detecting circadian mutants and/or clock modulators. Studies have used *in vitro* reporters to monitor alterations to the mammalian core clock following genetic (siRNA knockdown)^65,119,120^ or chemical modulation, including screening for novel small molecule modulators^91,92^ or characterize the effects of compounds on circadian parameters.^93,94,150^ To date, reporters have been instrumental in the identification and characterization of several small molecule clock modulators that interact with core and non-core clock proteins (*e.g.*, BMAL1, CRY1/2, REV-ERBs, RORs) through screens,^151,152^ or in evaluation of their activities, respectively.^150,153^

Similarly to their *in*/*ex vivo* counterparts, for *in vitro* reporters, the signal from a promoter/protein reporter-bearing cell line is used as a surrogate for and to monitor the transcriptional or translational activity of the desired circadian gene over several days. While most of the circadian promoter reporters described here are based on murine sequences, they have also been employed in *in vitro* cell culture models derived from humans. Genetic similarity between murine and human genomic sequences has facilitated the use of this strategy at the transcriptional level. However, any variation(s) in circadian behavior due to species-specific downstream regulation remain to be studied in depth. Most reporters used to monitor circadian oscillations track promoter activity using luciferase. While our group has recently described the generation of human-sequence based reporters for *PER2*^154^ and *BMAL1*,^155^ the murine iterations have been most commonly used in various cell models to track the oscillations of and any alterations to circadian rhythms, including in response to perturbations and/or disease.

In addition to the screening and effects-characterization applications described above, *in vitro* reporters have been useful for understanding circadian oscillations and the roles of various components. For example, the use of *in vitro* reporter models tracking *Bmal1-Luc* oscillations in NIH3T3 (murine) fibroblasts was crucial in finding that oscillations were self-sustained and persisted following cell division.^30^ A *Bmal1-Luc* reporter introduced to immortalized primary murine fibroblasts^29^ provided insights into the presence of *Bmal1* oscillations in peripheral cells and the roles of the ROR/REV/*Bmal1* feedback loop. Each of these reporters used different sections of the promoter sequence and varying lengths that were cloned from the murine genomic sequence. Other studies have also utilized *Bmal1*-based reporters developed using different sections of the *Bmal1* promoter, including NIH3T3^28,156^ and the human bone osteosarcoma cell line U2OS,^31^ among others.

Similarly, several murine *Per2* promoter-reporters have been developed using varying lengths of the promoter sequence and/or expressing different luciferase genes.^29,59,134,157,158^*Per2*-based studies performed in immortalized cell lines (NIH3T3 and MCF10A, respectively) substantiated the presence of robust circadian gene expression in peripheral tissues.^74,159^ Zhang *et al.* conducted a genome-wide screen using RNA interference (RNAi) and measured the gene expression effects using *Bmal1* and Per2 promoter-reporters.^89^ This study demonstrated the presence of ∼1000 genes that exhibited strong circadian phenotypes in U2OS cells. Since, U2OS cells (bearing murine sequence-based reporters) have been used as the standard *in vitro* model to track circadian oscillations for understanding fundamental aspects of the core clock,^160,161^ and evaluating effects of entities on circadian rhythms.^162–164^ In recent years, fluorescent reporters have also been developed, including to track the interactions of the CRY1 protein.^28,69^ These are described further at the end of this section. Table 3 summarizes promoter- and protein-based reporters that have been used to study circadian oscillations and/or proteins in various *in vitro* models.

**Table 3:** Use of circadian reporters to track core clock oscillations in *in vitro* models

Component	Promoter/protein	Reporter entity	Cell line(s)	Citation(s)
Murine *Per1*	Promoter	Firefly luciferase	Rat-1 fibroblasts	165
Human PER1	Protein	Firefly luciferase	U2OS	31
Human *PER2*	Promoter	Firefly luciferase	U2OS	154
Human PER2	Protein	Firefly luciferase	U2OS	31
		mScarlet	U2OS, HCT-116	69
Murine *Per2*	Promoter	Enhanced luciferase and firefly luciferase	NIH3T3	134
		Firefly luciferase	Rat-1 fibroblasts	59
		Firefly luciferase	Immortalized primary murine fibroblasts	29
			U2OS	119
		Firefly luciferase	NIH-3T3-L1 adipocytes, MMH-D3 mouse hepatocytes	131
Murine Per2	Protein	EGFP	NIH3T3	28
Human *BMAL1*	Promoter	Firefly luciferase	U2OS	155
Murine *Bmal1*	Promoter	Firefly luciferase	Immortalized primary murine fibroblasts	29
			U2OS	31 and 89
			NIH3T3 and Rat-1	30
			NIH3T3	156 and 166–168
Murine Bmal1	Protein	EGFP/RFP	NIH3T3	28
Murine Clock	Protein	EGFP/RFP	NIH3T3	28
Murine *Cry1*	Promoter	Firefly luciferase	NIH3T3	169 and 170
Murine Cry1	Protein	RFP	NIH3T3	28
Human CRY1	Protein	mClover3	U2OS, HCT-116	69

In addition to using *in vitro* luciferase reporter systems to understand the fundamentals of the core clock, reporter cell lines have been used to study implications of the circadian clock in disease. *In vitro* reporters have been employed in disease models to monitor changes in the rhythmic expression of circadian components.^123,171^ Our group has previously used luciferase reporters to demonstrate the presence of rhythms in low-malignancy breast cancer cells,^32^ and the alterations to these rhythms across cancer progression.^33^*In vitro* cancer models expressing FBXL6- and BMAL1-driven luciferase reporters have been used to study the role of oncogenic genes like MYC in disrupting the molecular core clock.^172,173^*In vitro Bmal1-Luc* promoter reporters have also been employed to investigate the effect of Ras-induced deregulation of the circadian clock in human skin and colon cancer cell lines.^174^ Another study engineered human leukemic T-cells to express crustacean *Gaussia* luciferase (Gluc) under clock-driven response elements, and monitored for rhythmic secretion of Gluc.^175^ These engineered T-cells were then injected into NSG mice to study the transcriptional dynamics of the leukemic cells *in vivo.* Interestingly, the rhythmically oscillating T-cells decoupled following transplantation and were able to re-synchronize, possibly to the murine master clock, the mechanism of which remains unclear. *In vitro* reporter models have also been used to assess and investigate the effects of small molecules on circadian rhythms in disease models and cancer.^34,35,163,164,176^

Reporters have also been employed to elucidate the response of the core clock to chemotherapeutic and chemo-assistive drugs,^122,177^ and the development of a chronogenetic drug delivery system.^178^ By using a combination of reporters, we can track the effect(s) of modulating clock-driven components and the downstream processes that can result in disease progression. Table 4 documents *in vitro* luciferase reporters that have been employed for the study of circadian rhythms in the context of disease, particularly cancer.

**Table 4:** Luciferase-based promoter reporters for monitoring circadian rhythm changes in *in vitro* cancer models^a^

Component	Disease model	Cell line(s)	Ref.
*mBmal1 (only)*	Colorectal Carcinoma	U2OS (model control cell line); human colon cancer cell lines: HTs; 29, RKO, SW480, LIM1215, Caco-2, and HTC-116	174
	Hepatocellular Carcinoma (HCC)	Human HCC cell lines HepG2, Hep3B, and Huh7	179
	Hepatocellular Carcinoma (HCC)	Murine HCC cell line Hepa-1c1c7	180
	Neuroblastoma	U2OS (model control cell line); murine hepatocellular carcinoma cell line (mHCC) 3-4; MycN-amplified Neuroblastoma cell lines: Shep N-MYC-ER, SKNAS, and N-MYC-ER	172
*mPer2 (only)*	Renal Cell Carcinoma (RCC)	Human RCC cell lines: A704, ACHN, 786-O, A498, 769-P, and Caki-2	181
	Metabolic dysfunction	Human choriocarcinoma cell line JEG3	182
*mPer2*, *mBmal1*	Colorectal Carcinoma	HepG2 (model control cell line); human colon cancer cell lines: SW480, SW620	183
	Breast Cancer	Human breast cancer cell lines: MCF7, MDA-MB-231	32 and 184
	Breast Cancer	Human breast epithelial cell lines (varying malignancy): MCF10A, MCF10AT.Cl2, MCF10Ca1h, and MCF10Ca1a	33
	Melanoma	Murine melanoma cell line B16	185

a All reporters use firefly luciferase.

The expression patterns of circadian genes in health and disease have been studied/tracked extensively using luciferase-based transcriptional promoter reporters, but in order to quantitatively evaluate the locations and interactions of circadian proteins and their complexes, protein-based reporters are essential. While the outputs from luminescent reporters are quite sensitive and can reflect subtle variations, their intensities are very low and are mostly used only to provide information at the population level. In a fairly recent study using U2OS cells, endogenous PER1 and PER2 genes were fused with luciferase using CRISPR knock-in (KI).^31^ Using the PER1/2-Luc KI cell lines, alterations in PER1/2 levels in peripheral cells were found when CRY proteins were knocked out. While still useful, it is not enough to help us understand the dynamics at subcellular levels.

To gain insights into the spatiotemporal dynamics of core clock proteins within cells, fluorescent protein reporters have been developed.^28,69^ They have been critical to understanding the roles of PER2:CRY1 as repressors^69^ and provide critical insights into BMAL1:CLOCK interactions and their crosstalk with PER2:CRY1 complex.^28^ Since these reporters use fluorescent proteins, they cannot effectively monitor subtle changes in translational expression over extended periods due to photobleaching and phototoxicity.^186^ Nonetheless, the tracking of circadian expression and location of core proteins has been enabled by tagging with fluorescent entities, including GFP, RFP, mScarlet, and mClover. PER2 and CRY1 protein reporters generated using the CRISPR knock-in strategy allowed for the monitoring of real-time interaction dynamics of these negative regulators using mScarlet and mClover reporters in U2OS cells.^69^ Using fluorescence imaging in live single cells, it was also revealed that the CRY1 protein was mostly nuclear and almost 5–10 fold more abundant than PER2. Separately, Koch *et al.* developed Bmal1 and Clock fusion proteins with EGFP and RFP, respectively, in murine 3T3 fibroblasts, to understand the quantitative biology behind BMAL1:CLOCK interactions and crosstalk with the PER2:CRY1 complex.^28^ Recently, a lentiviral construct expressing a truncated *Nr1d1*/REV-ERBα gene upstream of a destabilized Venus fluorescent protein was reported.^187^ This construct was adapted from a full-length REV-ERBα-Venus construct, published by Nagoshi *et al*.,^30^ to aid in increased transfection efficiency and ease of generating stable cell lines. Although fluorescent reporters are generally not suitable for long-term tracking, the truncated REV-ERBα-Venus construct was successfully used to track REV-ERBα oscillations over a period of 60 hours and to compute circadian parameters at the single-cell level.

## Summary and future research

The mammalian circadian machinery comprises a complex network of transcriptional activators and repressors. The regulation of the core clock is governed extensively by post-translational modifications.^188–190^ The roles of individual circadian components have been elucidated using molecular studies and standard biochemical approaches like western blotting and RT-PCR, and newer ones like next-generation sequencing (NGS). In contrast, fluorescent and bioluminescent reporters can provide detailed, time-resolved, complementary information regarding circadian gene expression in a high-throughput manner. This review documents numerous studies that developed and/or applied reporters to track the circadian behavior of individual components in mammals and cells derived from them. The current circadian reporter toolkit includes transcriptional and translational reporters to track the core (and a few non-core) clock components in *in*/*ex vivo* as well as *in vitro* models. Using reporters, researchers have been able to monitor the oscillations of core clock components in the SCN, as well as in peripheral tissues and cells. Fluorescent protein reporters have also been crucial in visualizing the spatio-temporal localization of core and non-core clock components. Circadian reporters have also provided visualizations and quantifications of alterations to oscillations in various disease models and evaluations of entities that are capable of affecting circadian rhythms.

Apart from understanding the molecular basis of clock components in health and disease, reporters have also been widely used as a screening tool to identify and/or evaluate entities that alter circadian rhythms in cells. With the growing interest in chronopharmacology, the identification of small molecule modulators that target the circadian clock and timing of therapeutic dosing has taken center stage.^24,191–195^ Circadian reporters provide highly detailed, quantitative information that can facilitate compound comparisons for evaluating leads and accurately indicate where in circadian timing particular cells are for dosing studies. Their use is essential to the connection between therapeutics and circadian rhythms.

While the current toolkit of reporters has had a significant role in discerning the dynamic function of clock components, they have largely been constrained to single-component assessments either at transcriptional or translational levels. Additionally, there is limited knowledge regarding differing roles of and compensatory effects among the paralogs/isoforms and numerous post-transcriptional and post-translational mechanisms that govern the clock.^189,190,196–198^ Some studies have used a combination of bioluminescent and fluorescent reporters to track the components across the different levels of expression.^88,199^ However, the primary limitation of using fluorescent reporter models is their diminished ability to allow long-term monitoring and lower sensitivity due to autofluorescence. There is a great need to expand the current reporter toolkit to include bioluminescent reporters that can allow the simultaneous tracking of multiple components and/or promoter activity and protein oscillations within the same cell line in real-time. Multiplexing of bioluminescent reporters would facilitate the correlation of post-translational changes to mRNA level oscillations within the same cell, in greater depth. This is also especially important in disease models, *in*/*ex vivo* and *in vitro*.

Another area that should be addressed is the limited use of humanized models to track physiological roles and mechanisms of circadian variation. Murine reporter models have been successfully used to understand the inner workings of the mammalian core clock. Previous reports have also highlighted the presence of conserved sequences across murine and human sequences.^200^ However, there has been increasing evidence that suggests differing physiological properties in peripheral cells of human origin.^201–203^ As a solution, the generation of reporters governed by human-derived circadian promoters for use in human-derived *in vitro* models, and the use of humanized mouse models, is suggested for further study of human clock regulatory machinery. This will contribute to a better understanding of circadian mechanisms in humans, elucidation of the role of circadian clock alterations in disease-related processes, and assist in the discovery of potential chronotherapies and therapeutic targets.

## Conflicts of interest

There are no conflicts to declare.

## Data Availability

No primary research results have been included, and no new data were generated or analyzed as part of this review.
